# Antimicrobial Resistance and Antimicrobial Activity of *Staphylococcus lugdunensis* Obtained from Two Spanish Hospitals

**DOI:** 10.3390/microorganisms10081480

**Published:** 2022-07-22

**Authors:** Rosa Fernández-Fernández, Carmen Lozano, Laura Ruiz-Ripa, Beatriz Robredo, José Manuel Azcona-Gutiérrez, Carla Andrea Alonso, Carmen Aspiroz, Myriam Zarazaga, Carmen Torres

**Affiliations:** 1 Área Bioquímica y Biología Molecular, OneHealth-UR Research Group, Universidad de La Rioja, 26006 Logroño, Spain; rosa.fernandez.1995@gmail.com (R.F.-F.); laura_ruiz_10@hotmail.com (L.R.-R.); myriam.zarazaga@unirioja.es (M.Z.); 2 Área Didáctica de las Ciencias Experimentales, OneHealth-UR Research Group, Universidad de La Rioja, 26006 Logroño, Spain; beatriz.robredo@unirioja.es; 3 Servicio de Microbiología, Hospital San Pedro, 26006 Logroño, Spain; jmazcona@riojasalud.es (J.M.A.-G.); caalonso@riojasalud.es (C.A.A.); 4 Servicio de Microbiología, Hospital Royo Villanova, 50015 Zaragoza, Spain; caspirozs@gmail.com

**Keywords:** *S. lugdunensis*, coagulase-negative-staphylococci, antibiotic resistance, bacteriocins

## Abstract

*Staphylococcus lugdunensis* is a coagulase-negative-staphylococci (CoNS) that lately has gained special attention in public health as a human pathogen and also as a bacteriocin-producer bacteria. In this study, we characterized 56 *S. lugdunensis* isolates recovered from human samples in two Spanish hospitals. Antimicrobial susceptibility testing was performed and antimicrobial resistance and virulence genotypes were determined. Antimicrobial activity (AA) production was evaluated by the *spot-on-lawn* method against 37 indicator bacteria, including multidrug-resistant (MDR) isolates, and the presence of the *lug*D gene coding for lugdunin bacteriocin was analyzed by PCR. The antibiotic resistance detected was as follows (% resistance/genes detected): penicillin (44.6%/*bla*Z), oxacillin (1.8%/*mecA* on SCC*mec*-V), erythromycin-clindamycin inducible (7.1%/*erm*(C), *msrA*), tetracycline (5.3%/*tetK*), gentamicin and/or tobramycin (3.6%/*ant*(4′)-Ia, *acc*(6′)-*aph*(2″)), and fosfomycin (21.4%). A MDR phenotype was detected in 5% of isolates. Twenty-one of the *S. lugdunensis* isolates showed susceptibility to all 20 antibiotics tested (37.5%). The screening for AA revealed 23 antimicrobial producer (AP) isolates with relevant inhibition against coagulase-positive-staphylococci (CoPS), including both methicillin-susceptible and –resistant *S. aureus*. The *lug*D gene was detected in 84% of the 56 *S. lugdunensis* isolates. All of the AP *S. lugdunensis* isolates (*n* = 23) carried the *lug*D gene and it was also detected in 24 of the non-AP isolates, suggesting different gene expression levels. One of the AP isolates stood out due to its high antimicrobial activity against more than 70% of the indicator bacteria tested, so it will be further characterized at genomic and proteomic level.

## 1. Introduction

Coagulase-negative staphylococcal species (CoNS) are commensal bacteria in humans and animals. *Staphylococcus lugdunensis* belongs to the CoNS group, and it is part of the normal human skin microbiota that also has been found in the nasal cavity [1]. Some CoNS, such as *S. lugdunensis*, are considered as significant opportunistic pathogens due to their implication in different human infections [2], going from skin and soft tissue infections (SSTI) to invasive diseases such as infective endocarditis, bone and joint infections, prosthetic joint-infections, vascular catheter-related infections and abscesses, among others [1,2].

Notably, most bacterial infections are caused by pathogens from the human microbiota. Individuals colonized with multidrug resistant (MDR) microorganisms are exposed to higher risks of invasive infections with more difficult treatment in surgery or immunosuppression cases [3]. In this sense, the current antibiotic resistance problem represents an important health emergency, being the major cause of morbidity and mortality associated with infections worldwide including in developed countries [4,5].

Fortunately, *S. lugdunensis* usually remains susceptible to many antibiotics [6]. However, although *S. lugdunensis* strains (as other CoNS) lack many of the common virulence factors of *S. aureus,* other virulence mechanisms have been identified in this species [7]. In this respect, *S. lugdunensis* has lately been recognized as a pathogenic microorganism and should be considered between one of the most clinical relevant CoNS.

On the other hand, recent studies have reported that *S. lugdunensis* can produce a novel cyclic antimicrobial peptide named lugdunin, which is included in a new class of antibacterials due to its non-ribosomal synthesis. Lugdunin displays a potent antimicrobial activity against a wide range of Gram-positive bacteria including methicillin resistant *S. aureus* (MRSA) [3]. In this sense, human microbiota should be considered as a source for new antimicrobial substances [8,9].

The objective of this study was to characterize a collection of *S. lugdunensis* isolates recovered at two hospitals located in different Spanish regions and determine the phenotypes and genotypes of antibiotic resistance, the virulence content, and the production of antimicrobial compounds against a wide selection of indicator bacteria (different genera/species), including MDR microorganisms.

## 2. Material and Methods

### 2.1. Bacterial Collection

This retrospective study included 56 *S. lugdunensis* isolates recovered during a five-year period (2013–2018) from patients of two Spanish hospitals: 48 isolates from Hospital San Pedro (HSP) of Logroño and eight from Hospital Royo Villanova (HRV) of Zaragoza. These isolates were obtained from the following type of samples: skin and soft-tissue infections (SSTI, *n* = 23), catheter (*n* = 13), blood (*n* = 8), urine (*n* = 7), genital exudates (*n* = 4), and epidemiological samples (*n* = 1) (Supplementary Table S1). Antimicrobial resistance, virulence content and bacteriocin production capacity were characterized in these isolates.

### 2.2. Antimicrobial Resistance Phenotype and Genotype

The susceptibility testing for antimicrobial agents was performed by the commercialized broth microdilution method (Microscan, Beckman Coulter, Brea, CA, USA). Twenty antimicrobial agents were tested: penicillin, oxacillin, cefoxitin, ceftaroline, gentamicin, tobramycin, ciprofloxacin, levofloxacin, erythromycin, clindamycin, pristinamycin, linezolid, fosfomycin, mupirocin, tetracycline, trimethoprim-sulfamethoxazole, vancomycin, teicoplanin, quinupristin/dalfopristin, and daptomycin. The antimicrobial resistance phenotype was evaluated according to the European Committee on Antimicrobial Susceptibility Testing criteria [10].

Based on the resistance phenotype, the presence of the following antimicrobial resistance genes was investigated by PCR: *bla*Z, *mec*A, *mec*C, *tet*(L), *tet*(K), *tet*(M), *msr*(A), *erm*(A), *erm*(B), *erm*(C), *acc*(6′)-*aph*(2″), *ant*(4′)-Ia, *mup*(A)*,* and *mup*(B) [11,12]. The methicillin resistant *S. lugdunensis* isolates were subjected to SCC*mec*-typing [13].

### 2.3. Virulence Content

The presence of the following virulence genes was tested by PCR: leukocidin genes (*luk*SF-PV, *luk*M, *luk*ED, and *luk*PQ), the toxic shock syndrome toxin 1 (*tst*), and the exfoliative toxins A, B, D (*eta, etb,* and *etd*) [12].

Positive and negative control strains from the collection of the Universidad de La Rioja were included in all PCR assays for antimicrobial resistance genotype and virulence content.

### 2.4. Antimicrobial Activity

The screening of antimicrobial activity (AA) production was performed for the 56 *S. lugdunensis* isolates by the *spot-on-lawn* method using 37 indicator bacteria (including diverse genera and species, as well as MDR bacteria and relevant pathogens). The characteristics of the indicator bacteria are included in Supplementary Table S2. Bacteria were grown in brain heart infusion (BHI) agar (Condalab, Spain) for 24 h at 37 °C. In order to prepare test plates, 5 mL of sterile semisolid Tryptic Soy Broth (SS-TSB) (BD, Difco, France) supplemented with 0.3% yeast extract and 0.7% agar was maintained at 45 °C, inoculated with 10 µL of a 0.5 MacFarland BHI broth dilution of each indicator strain and poured and spread as a lawn onto yeast extract-supplemented solid Tryptic Soy Agar (TSA) (BD, Difco, France) plates. A single colony of each *S. lugdunensis* isolate to be tested for AA production was transferred with a sterile toothpick to the agar plate seeded with the indicator. Plates were incubated at 37 °C for 24 h to evaluate the halo of inhibition growth (in mm) [14]. Isolates were considered antimicrobial producers (AP) when they showed a clear inhibition zone against at least one of the 37 indicator isolates.

Moreover, the presence of the *lug*D gene was taken as a reference to identify the genetic cluster associated with the production of the non-ribosomal peptide (NRP), lugdunin (GenBank accession number NC_017353.1). For that, the *lug*D amplicon (189 pb) was identified using the following primers and PCR conditions: F-TTCGGGAACTACTGGAATGC (Tm = 60.1 °C), R-AAATGCAATGTCCCTCCAAC (Tm = 59.8 °C); 1 cycle at 94 °C for 7 min, 30 cycles at 94 °C for 1 min, 57 °C for 1 min, 72 °C for 1 min, and finally 72 °C for 10 min [15]. Subsequently, *lug*D amplicons were confirmed by Sanger sequencing.

### 2.5. Statistical Analysis

The Pearson’s chi-square test was used to explore significant differences between the isolates tested. Analyses were carried out using SPSS statistical software version 26.0 (IBM^®^, SPSS Inc., Chicago, IL, USA) and significance was set at *p* ≤ 0.05.

## 3. Results

*S. lugdunensis* represented 2.4% of the infections caused by CoNS in the two tested hospitals. The collection of 56 *S. lugdunensis* isolates included in this study represented approximately 35% of the total *S. lugdunensis* recovered in the period 2013–2018 in those hospitals, (the remaining isolates were not maintained and they could not be analyzed); they were obtained from a wide diversity of origins (mostly implicated in infections, 42 out of the 56 total isolates): SSTI (41%), catheter (23%), blood (14.3%) urine (12.5%), genital exudate (7.1%) and epidemiological samples (1.8%) (Table 1).

### 3.1. Phenotype and Genotype of Antimicrobial Resistance

The phenotypes and genotypes of antimicrobial resistance of the 56 *S. lugdunensis* isolates included in this study are shown in Table 1. In this respect, 62.5% of the isolates showed resistance to at least one of the antimicrobial agents tested: penicillin (44.6%), oxacillin (1.8%), fosfomycin (21.4%), erythromycin-clindamycin (7.1%), tetracycline (5.3%), tobramycin (3.5%), gentamicin (1.8%) and mupirocin (1.8%). No isolate showed resistance for the remaining tested antibiotics. Three isolates (5%) were MDR (showing resistance to three or more families of antimicrobial agents) (Table 1). Focusing on the sample origin, the rates of resistant isolates (for at least one tested antibiotic) were as follows: epidemiological sample (100%, one isolate), blood (87.5%), urine (71.4%), SSTI (65.2%), genital exudate (50%) and catheter (42.9%). All 25 penicillin-resistant isolates carried the *bla*Z gene; in addition, genes implicated in the macrolide/lincosamide [*erm*(C), *msr*(A)] and aminoglycoside [*aac*(6′)-*aph*(2″), *ant*(4′)-Ia] resistances were also detected. Tetracycline and mupirocin resistances were rarely found in our collection and were mediated by the *tet*(K) and *mup*(A) genes, respectively. With respect to the methicillin resistance, it was confirmed that the *S. lugdunensis* strain C9897 carried the *mec*A gene within the SCC*mec* type V element (Table 2).

Moreover, none out of the 56 isolates carried any of the virulence genes studied.

### 3.2. Antimicrobial Activity

Twenty-three antimicrobial producer (AP) isolates (41%) with activity against at least one of the 37 indicator bacteria tested were found in this study. They were identified by the *spot-on-lawn* method, including indicator bacteria of the following relevant genera (number of isolates): staphylococci (26), enterococci (7), and *Listeria* (1), among others (Table 3 and Supplementary Tables S2 and S3). The AP isolates were recovered mainly from samples of SSTI (43%), but also from samples of catheter, urine and blood (Table 1). The 23 AP isolates could be differentiated in the following categories: (i) nine isolates showed a broad interspecific activity (InterA-AP), because indicators of at least two different genera were inhibited by the producer isolate; (ii) 12 AP isolates showed broad intraspecific activity (IntraA-AP) because the activity was only detected against indicator bacteria of the same genera as the producer one (*Staphylococcus*), but was of several species; (iii) two isolates considered as moderate antimicrobial producers due to their reduced (RA-AP) spectrum of activity (Table 3).

**Figure 1:**
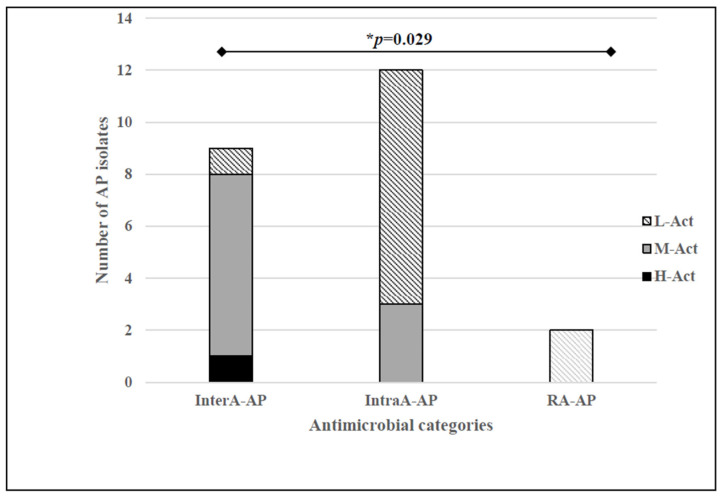
Number of antimicrobial producer (AP) isolates of each established antimicrobial categories (InterA-AP, IntraA-AP and RA-AP) that present high, medium and low antimicrobial activity (H-Act, M-Act and L-Act, respectively). * Statistically significant differences were observed (*p* ≤ 0.05).

Moreover, three levels of antimicrobial activity were established based on the percentage of indicator bacteria inhibited by each AP *S. lugdunensis* isolate: high activity (H-Act, activity against >70% of the indicator bacteria tested), medium activity (M-Act, 20–70%), and low activity (L-Act, <20%) (Figure 1).

**Figure 2:**
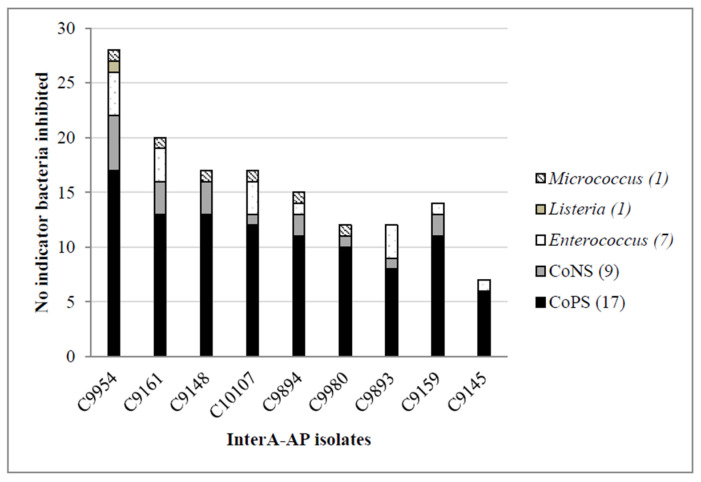
Antimicrobial profile summary of the 9 high antimicrobial-producers *S. lugdunensis* isolates with a broad Interespecific Activity (InterA-AP). Abbreviations: CoPS, coagulase-positive *Staphylococcus*; CoNS, coagulase-negative *Staphylococcus*.

The 9 *S. lugdunensis* isolates classified in the broad InterA-AP category were recovered from blood (*n* = 1), catheter (*n* = 4), SSTI (*n* = 2) and urine (*n* = 2). The antimicrobial profiles of these isolates are summarized in Figure 2, showing an interesting inhibition capacity against more than three relevant indicator bacteria species such as coagulase-positive staphylococci (CoPS), CoNS, *Enterococcus*, *Micrococcus luteus* and *Listeria monocytogenes*. In terms of antimicrobial activity levels, only one InterA-AP isolate (C9954) showed high activity against 76% of the indicators tested and the rest showed medium (*n* = 7) or low (*n* = 1) antimicrobial activity. As for the IntraA-AP and RA-AP isolates, only three IntraA-AP *S. lugdunensis* isolates were considered medium producers because they inhibit 30% of the indicator bacteria tested, while the others only showed antimicrobial activity against less than 5% of the indicators (Figure 1).

Moreover, PCR and sequencing analysis confirmed that all 23 of the AP isolates, and 24 of the 33 non-AP isolates carried the gene *lug*D, which codes for a protein implicated in the synthesis of the NRP, lugdunin (Table 1). Only nine *S. lugdunensis* isolates were negative for antimicrobial activity by the *spot-on-lawn* method and did not carry the *lug*D gene.

### 3.3. Antibiotic Resistance Phenotype versus Antimicrobial Activity

**Figure 3:**
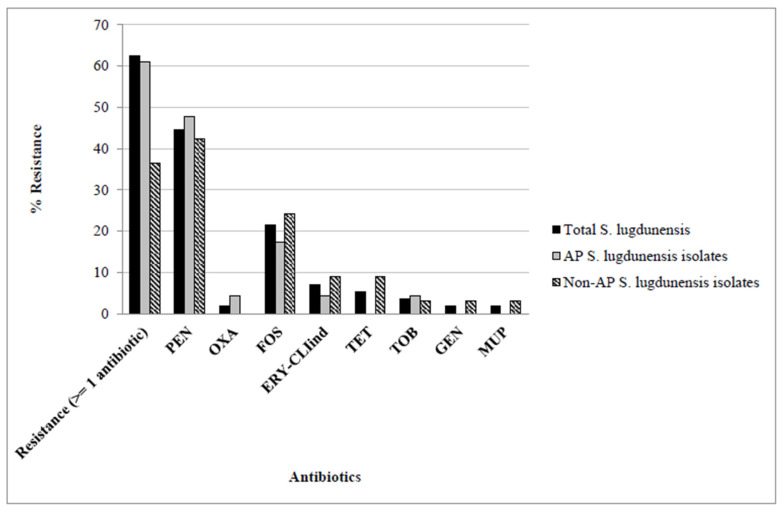
Antimicrobial resistance phenotype of the 56 *S. lugdunensis* isolates versus the 23 AP and the 33 non-AP *S. lugdunensis* isolates. Abbreviations: PEN, penicillin; OXA, oxacillin; FOS, Fosfomycin; ERY-CLIind, erythromycin-clindamycin inducible; TET, tetracycline; TOB, tobramycin; GEN, gentamycin; MUP, mupirocin. Non-statistically significant differences (*p* ≤ 0.05) were observed.

The antimicrobial resistance phenotype of the 56 *S. lugdunensis* isolates compared to that of the 23 AP and the 33 Non-AP isolates is shown in Figure 3. Similar resistance rates were found when all *S. lugdunensis* or only AP isolates were considered (total%/AP%): in this sense, 62.9%/60.9% of the *S. lugdunensis* isolates showed resistance to at least one of the antimicrobials tested, and penicillin was the most frequently observed, with a rate of 44.6%/47.8%, followed by fosfomycin (21.4%/17.3%). The rate of resistance to oxacillin, tobramycin, erythromycin and clindamycin was lower (<5%). Focusing on Non-AP *S. lugdunensis* isolates, the antimicrobial resistance rate was lower (36.4%) and the following resistance percentages were detected: penicillin (42.4%), fosfomycin (24.2%), erythromycin-clindamycin and tetracycline (9.1%), and tobramycin, gentamicin and mupirocin (3%).

Based on the antimicrobial activity categories, Table 4 summarizes the origin, type of sample, antimicrobial resistance phenotype/genotype and bacteriocin genes of the 23 AP isolates. Focusing on InterA-AP isolates, 33% of them (*n* = 3) were susceptible to all the antimicrobials tested, including the two isolates with a higher inhibition profile (C9954 and C9161). However, four isolates showed resistance exclusively to penicillin, one isolate showed resistance to penicillin and fosfomycin, and the other was resistant to erythromycin-clindamycin^Inducible^. As for the antimicrobial resistance profile of IntraA-AP and RA-AP isolates, 42.8% of them showed susceptibility to all of the antimicrobials tested. Among the resistant isolates, four showed resistance exclusively to penicillin, two exclusively to fosfomycin, one isolate was resistant to penicillin and fosfomycin, and other one showed resistance to penicillin, oxacillin and tobramycin.

Non-statistically significant differences were found when comparing the origin of the isolates, the antimicrobial activity, and also the established categories (Inter-AP, Intra-AP and RA-AP), and their antimicrobial resistance phenotype. However, the correlation between categories of antimicrobial production and the antimicrobial activity levels revealed statistically significant values (*p* = 0.029) (Figure 1). Moreover, focusing on categories, the antimicrobial activity against *Enterococcus* and *Micrococcus* was also statistically significant (*p* = 0.034 and *p* = 0.046, respectively).

## 4. Discussion

*S. lugdunensis* is a component of the human microbiome and its role in a wide spectrum of diseases has been recently demonstrated [16]. It has been estimated that *S. lugdunensis* physiological colonization affect to the 30% to 50% of patients [17,18]. *S. lugdunensis* has low presence in human clinical samples, ranging from 0.5% to 9% in CoNS-positive samples [19,20]. However, recent studies have reported that the proportions of CoNS identified as *S. lugdunensis* and their isolation frequency have steadily increased, although susceptibility rates were not substantively modified during the studied time [21].

In our study, *S. lugdunensis* represented approximately 2.4% of the total CoNS isolated from several origin samples during a five-year period, which reveal a low implication rate with respect to the total CoNS. A relevant percentage of the samples were obtained from SSTI (41%), followed by those associated with catheters (23%). Moreover, isolates obtained from blood cultures (14%), urine (13%), genital exudates (7%) or epidemiological isolates (2%) were also detected. Although many of the *S. lugdunensis* isolates are not especially pathogenic and commonly act as colonizer bacteria, these CoNS should not be undervalued.

*S. lugdunensis* has been referred to in the literature as a remarkably susceptible CoNS specie for most of antibiotics [1]. In this study, 21 out of the 56 isolates (37.5%) were susceptible to all groups of antibiotics tested. Different penicillin resistance rates have been detected among *S. lugdunensis* isolates worldwide, from 15–25% in Sweden and Denmark [6,22,23] to 87% in Taiwan [24]. Our penicillin resistance results (44.6%) were similar to those found in previous studies carried out in the USA [21,25]. Significantly, a perfect concordance between resistance phenotype/genotype for penicillin was detected in our study using Microscan. This resistance was mediated in 100% of the penicillin resistant isolates by the expression of the *bla*Z gene. However, other studies have noted a phenotype-genotype discrepancy in relation to penicillin resistance detection when other commercial microdilution methods were used [26].

As for methicillin resistance, only one *S. lugdunensis* isolate was identified as methicillin-resistant which carried the *mec*A gene. Similar results were published by [25], revealing that 3% of the 36 isolates tested were oxacillin resistant and displayed the *mecA* gene. Although there is incomplete information about the SCC*mec* types present in methicillin-resistant *S. lugdunensis* isolates, it has been reported some isolates carriers of elements that were variants of SCC*mec* type V [27]. Therefore, a comprehensive analysis of the SCC*mec* types is required to better understand the acquisition and spread of resistance to beta-lactams [1]. It is to highlight the low beta-lactam resistance detected in the *S. lugdunensis* studied isolates within more than half of the isolates were susceptible to penicillin and oxacillin resistance was rarely detected. As already suggested by others [21], the possibility of using narrow-spectrum beta-lactam agents must be strongly considered in the treatment of infections for this CoNS species.

Resistance to macrolide/lincosamide antibiotics, such as erythromycin and clindamycin, is overall very low [23,25], representing only a 7.1% of the total isolates tested in our work. Moreover, aminoglycoside, tetracycline and mupirocin resistances were also detected, but in low percentages.

The high frequency of fosfomycin resistance detected in our collection of *S. lugdunensis* isolates (21.4%) is of interest; very few studies focused on this antimicrobial agent, although high resistance levels have been reported in some of them (>50%) [28].

As for the virulence content of CoNS, *S. lugdunensis* has been recognized as a CoNS species with a considerable pathogenic potential [7]. Our isolates lacked all the virulence genes tested, previously described in *S. aureus* as being more associated with this species.

Antimicrobial resistance is becoming a severe public health problem and CoNS species deserve special attention due to their significant impact on the clinical and food fields. A better understanding of the processes governing bacterial fitness, competition, and bacteria dissemination is needed. In this sense, it is well known that human skin is populated by a complex microbiota [29,30] that protect us from pathogen colonization thanks to the release of specific antimicrobial peptides termed bacteriocins. *S. lugdunensis* usually acts as a human skin commensal, and recent studies highlight this specie due to its ability to produce lugdunin, a novel antibiotic compound that inhibits the growth of *S. aureus*, other Gram-positive bacteria, and even vancomycin-resistant enterococci [3].

In this study, 23 *S. lugdunensis* AP isolates were identified, differentiating between isolates with broad InterA-AP (*n* = 9) and those with IntraA-AP or RA-AP (*n* = 12 and *n* = 2, respectively). It is worth highlighting the 9 AP isolates with high antimicrobial activity against CoPS, relevant indicator bacteria such as MSSA and MRSA, *Enterococcus vanA/vanB2*, *Micrococcus luteus* and *Listeria monocytogenes*. One of these AP isolates (C9954) showed high inhibitory activity against more than 70% of the indicator bacteria*,* including MDR, so it will be an interesting candidate for a further in-depth characterization.

In addition, PCR and sequencing analysis confirmed the presence of *lug*D in 86% of the *S. lugdunensis* isolates studied. This gene is the precursor of lugdunin bacteriocin and conforms the NRP operon with the other four genes named *lug*A, B, C, and D. Zipperer et al. described in 2016 that this NRP operon was found in all *S. lugdunensis* genomes available in the databases. In the present study, the *lug*D gene was detected in all the 23 AP-positive isolates, but 24 of the 33 non-AP isolates were positive for the *lug*D gene. The lack of *lug*D in the other 9 non-AP *S. lugdunensis* isolates could be due to mutations on the primer region. In this respect, further genomic studies will be carried out in order to confirm the presence of the complete lugdunin operon in the tested isolates and to analyse the differences with those previously described.

In conclusion, in the present study, the 37.5% of *S. lugdunensis* isolates were susceptible to all tested antibiotics. More than half of the isolates were penicillin susceptible and only one was identified as methicillin-resistant. The low beta-lactam resistance detected in the *S. lugdunensis* studied isolates corroborates the possibility of using narrow-spectrum beta-lactam agents in the treatment of *S. lugdunensis* infections.

Twenty-three *S. lugduunensis* isolates showed antimicrobial activity, nine of them with high activity against CoPS, and one isolate with high inhibitory activity against more than 70% of the indicator bacteria. Its role in the modulation of microbiota in which this species is present seems to be of great relevance. Finally, most of the isolates contained the gene *lug*D, although this gene was not identified in 9 isolates. The relation among the presence/expression of this operon and the antimicrobial activity of *S. lugdunensis* isolates should be analyzed in the future.

## Figures and Tables

**Table 1:** Origin, antimicrobial resistance phenotype and genotype, antimicrobial activity production (AP) and bacteriocin genes of the 56 *S. lugdunensis* isolates included in this study.

Origin ^a^	Number of Isolates	Antimicrobial Resistance		Antimicrobial Activity	
		Phenotype ^b,c^	Genotype	AP ^d^	*lug*D
Catheter	5	Susceptible	NT	+	+
	1	Susceptible	NT	−	−
	1	Susceptible	NT	−	+
	1	Susceptible	NT	+	+
	1	PEN	*bla*Z	+	+
	3	PEN	*bla*Z	−	+
	1	FOS	NT	+	+
Epidemiological	1	PEN	*bla*Z	−	+
Blood	4	PEN	*bla*Z	−	+
	1	Susceptible	NT	−	+
	2	PEN	*bla*Z	+	+
	1	PEN−TET	*bla*Z, *tet*(K)	−	−
Genital Exudate	2	Susceptible	NT	−	+
	1	FOS	NT	−	−
	1	PEN−FOS	*bla*Z	−	+
SSTI	3	Susceptible	NT	−	−
	3	Susceptible	NT	−	+
	2	Susceptible	NT	+	+
	1	PEN	*bla*Z	−	−
	4	PEN	*bla*Z	+	+
	2	FOS	NT	−	−
	1	FOS	NT	+	+
	1	PEN−FOS	*bla*Z	+	+
	1	ERY−CLIind	*msr*(A)	+	+
	1	PEN− ERY−CLIind	*bla*Z, *erm*(C), *msr*(A)	−	+
	1	PEN−OXA−TOB	*bla*Z, *mec*A, *ant*(4′)−Ia	+	+
	1	ERY−CLIind−TET−FOS	*erm*(C), *msr*(A), *tet*(K)	−	+
	1	ERY−CLIind−MUP−FOS	*erm*(C), *msr*(A), *mup*(A)	−	+
	1	GEN−TOB−FOS	*ant*(4′)−Ia, *acc*(6′)−*aph*(2″)	−	+
Urine	1	Susceptible	NT	−	+
	1	Susceptible	NT	+	+
	1	PEN	*bla*Z	−	+
	1	PEN	*bla*Z	+	+
	1	FOS	NT	−	+
	1	PEN−TET	*bla*Z, *tet*(K)	−	+
	1	PEN−FOS	*bla*Z	+	+

^a^ Origin: SSTI: skin and soft tissue infection. ^b^ Abbrevations: PEN: penicillin; ERY: erythromycin; CLIind: clindamycin inducible; OXA: methicillin/cefoxitin; GEN: gentamicin; TOB: tobramycin; TET: tetracycline; FOS: fosfomycin; MUP: mupirocin. ^c^ Susceptible to all antimicrobial tested. ^d^ AP: antimicrobial producer; +/−: positive/negative; NT: non tested.

**Table 2:** Antimicrobial resistance phenotypic and genotypic correlation for all the antibiotics tested.

Antibiotic	No of Resistant Isolates	Antimicrobial Resistance Genes (No of Isolates)
Penicillin	25	*bla*Z (25)
Oxacillin	1	*mec*A included in SCC*mec*-V (1)
Fosfomycin	12	non studied
Erythromycin-Clindamycin inducible	4	*msr*(A) (1), *msr*(A) + *erm*(C) (3)
Tetracycline	3	*tet*(K) (3)
Tobramycin	2	*ant*(4′)-Ia (2)
Gentamicin	1	*acc*(6′)-*aph*(2″) (1)
Mupirocin	1	*mup*(A) (1)

**Table 3:** Antimicrobial activity of the 23 *S. lugdunensis* isolates characterized as bacteriocin producers against the 37 indicator bacteria.

Indicator Bacteria (nº Isolates) ^b^		Antimicrobial Activity of the Bacteriocin Producer Isolate against Indicator Bacteria (Number of Indicator Bacteria Inhibited)																						
		InterA-AP ^a^									IntraA-AP ^a^												RA-AP ^a^	
		C9954	C9161	C9148	C10107	C9894	C9980	C9893	C9159	C9145	C9892	C10052	C9890	C9911	C10343	C9142	C9146	C9147	C9151	C10320	C10341	C10511	C9897	C9342
**Gram +**	**MR-CoPS (6)**	6	3	3	4	2	1	-	4	1	1	2	2	-	-	1	1	1	-	-	-	-	-	-
	**MS-CoPS (11)**	11	10	10	8	9	9	8	7	5	9	8	8	1	1	-	-	-	-	-	-	-	-	-
	**CoNS (9)**	5	3	3	1	2	1	1	2	-	2	1	2	-	1	-	-	-	1	1	1	1	-	-
	***Enterococcus vanA/vanB2* (4)**	2	1	-	-	-	-	1	-	-	-	-	-	-	-	-	-	-	-	-	-	-	-	-
	**Other *enterococci* (3)**	2	2	-	3	1	-	2	1	1	-	-	-	-	-	-	-	-	-	-	-	-	1	-
	**Total *staphylococci* (26)**	22	16	16	13	13	11	9	13	6	12	11	12	1	2	1	1	1	1	1	1	1	-	-
	**Total *enterococci* (7)**	4	3	-	3	1	-	3	1	1	-	-	-	-	-	-	-	-	-	-	-	-	1	-
	***L. monocytogenes* (1)**	1	-	-	-	-	-	-	-	-	-	-	-	-	-	-	-	-	-	-	-	-	-	1
	***M. luteus* (1)**	1	1	1	1	1	1	-	-	-	-	-	-	-	-	-	-	-	-	-	-	-	-	-
**Gram −**	***E. coli* (1)**	-	-	-	-	-	-	-	-	-	-	-	-	-	-	-	-	-	-	-	-	-	-	-
	***P. aeruginosa* (1)**	-	-	-	-	-	-	-	-	-	-	-	-	-	-	-	-	-	-	-	-	-	-	-

^a^ Categories of antimicrobial activity: InterA-AP, Interespecific Activity (antimicrobial activity against different groups of bacteria belonging to different genera, in addition to staphylococci); IntraA-AP, Intraspecific Activity (antimicrobial activity against different species of staphylococci, but not against other genera); RA-AP, Reduced Activity (antimicrobial activity against one bacterial group, genera or species). ^b^ Abbreviations: MR, methicillin resistant; MS, methicillin susceptible; CoPS, coagulase-positive *Staphylococcus*; CoNS, coagulase-negative *Staphylococcus*.

**Table 4:** Origin, type of sample, antimicrobial resistance phenotype/genotype and bacteriocin genes of the 23 AP isolates based on the antimicrobial activity categories.

Antimicrobial Activity ^a^	Number of Isolates	Origin ^b,c^	Antimicrobial Resistance Phenotype ^b^	Antimicrobial Resistance Genotype ^b,d^	No of Isolates Carring *lugD*
InterA-AP	9	Blood^1^-Catheter^4^-SSTI^2^-Urine^2^	Susceptible^3^-PEN^5^-FOS^1^-(ERY- CLIind)^1^	*bla*Z^5^-*msr*(A)^1^	9
IntraA-AP	12	Blood^1^-Catheter^4^-SSTI^6^-Urine^1^	Susceptible^6^-PEN^5^-FOS^2^	*bla*Z ^5^	12
RA-AP	2	SSTI^2^	PEN^1^-FOS^1^-OXA^1^-TOB^1^	*bla*Z^1^, *mec*A^1^, *ant*(4′)(4‘’)^1^	2

^a^ Categories of antimicrobial activity: InterA-AP, Interespecific Activity (antimicrobial activity against different groups of bacteria belonging to different genera, in addition to staphylococci); IntraA-AP, Intraspecific Activity (antimicrobial activity against different species of staphylococci, but not against other genera); RA-AP, Reduced Activity (antimicrobial activity against one bacterial group). ^b^ A number in superscript indicates the total isolates with the indicated characteristic. ^c^ Origin: SSTI: skin and soft tissue infection. ^d^ Abbreviations: PEN: penicillin; OXA: methicillin/cefoxitin; FOS: fosfomycin; ERY- CLIind: erythromycin-clindamycin inducible; GEN: gentamicin; TOB: tobramycin.

## Supplementary Materials

The following supporting information can be downloaded at: https://www.mdpi.com/2076-2607/10/8/1480/s1: Table S1: Clinical characteristics of the *S. lugdunensis* isolates included in the study; Table S2: Characteristics of the 37 indicator bacteria used in this study for the screening of antimicrobial activity production in the collection of 56 *S. lugdunensis* isolates; Table S3: Antimicrobial activity profile of the 23 *S. lugdunensis* isolates characterized as antimicrobial producers against the 37 indicator bacteria.

## References

[1] Heilbronner S., Foster T.J. (2020). *Staphylococcus lugdunensis*: A Skin Commensal with Invasive Pathogenic Potential. Clin. Microbiol. Rev..

[2] Lebeurre J., Dahyot S., Diene S., Paulay A., Aubourg M., Argemi X., Giard J.-C., Tournier I., François P., Pestel-Caron M. (2019). Comparative Genome Analysis of *Staphylococcus lugdunensis* Shows Clonal Complex-Dependent Diversity of the Putative Virulence Factor, ess/Type VII Locus. Front. Microbiol..

[3] Zipperer A., Konnerth M.C., Laux C., Berscheid A., Janek D., Weidenmaier C., Burian M., Schilling N.A., Slavetinsky C., Marchal M. (2016). Human commensals producing a novel antibiotic impair pathogen colonization. Nature.

[4] Arias C.A., Murray B.E. (2009). Antibiotic-Resistant Bugs in the 21st Century—A Clinical Super-Challenge. N. Engl. J. Med..

[5] Laxminarayan R., Duse A., Wattal C., Zaidi A.K.M., Wertheim H.F.J., Sumpradit N., Vlieghe E., Hara G.L., Gould I.M., Goossens H. (2013). Antibiotic resistance—The need for global solutions. Lancet Infect. Dis..

[6] Taha L., Stegger M., Söderquist B. (2019). *Staphylococcus lugdunensis*: Antimicrobial susceptibility and optimal treatment options. Eur. J. Clin. Microbiol. Infect. Dis..

[7] França A., Gaio V., Lopes N., Melo L. (2021). Virulence Factors in Coagulase-Negative Staphylococci. Pathogens.

[8] Dobson A., Cotter P., Ross R., Hill C. (2012). Bacteriocin Production: A Probiotic Trait?. Appl. Environ. Microbiol..

[9] Kommineni S., Bretl D.J., Lam V., Chakraborty R., Hayward M., Simpson P.M., Cao Y., Bousounis P., Kristich C.J., Salzman N.H. (2015). Bacteriocin production augments niche competition by enterococci in the mammalian gastrointestinal tract. Nature.

[10] EUCAST The European Committee on Antimicrobial Susceptibility Testing. Breakpoint Tables for Interpretation of MICs and Zone Diameters. Version 12.0. 2022. http://www.eucast.org.

[11] Benito D., Lozano C., Gómez-Sanz E., Zarazaga M., Torres C. (2013). Detection of Methicillin-Susceptible Staphylococcus aureus ST398 and ST133 Strains in Gut Microbiota of Healthy Humans in Spain. Microb. Ecol..

[12] Ruiz-Ripa L., Gómez P., Alonso C.A., Camacho M.C., Ramiro Y., de la Puente J., Fernández-Fernández R., Quevedo M., Blanco J.M., Báguena G. (2020). Frequency and Characterization of Antimicrobial Resistance and Virulence Genes of Coagulase-Negative Staphylococci from Wild Birds in Spain. Detection of *tst*-Carrying *S. sciuri* Isolates. Microorganisms.

[13] Yeh C.-F., Chang S.-C., Cheng C.-W., Lin J.-F., Liu T.-P., Lu J.-J. (2016). Clinical Features, Outcomes, and Molecular Characteristics of Community- and Health Care-Associated *Staphylococcus lugdunensis* Infections. J. Clin. Microbiol..

[14] del Campo R., Tenorio C., Jiménez-Díaz R., Rubio C., Gómez-Lus R., Baquero F., Torres C. (2001). Bacteriocin Production in Van-comycin-Resistant and Vancomycin Susceptible Enterococcus Isolates of Different Origins. Antimicrob. Agents Chemother..

[15] Fernández-Fernández R., Lozano C., Eguizábal P., Ruiz-Ripa L., Martínez-Álvarez S., Abdullahi I.N., Zarazaga M., Torres C. (2022). Bacteriocin-Like Inhibitory Substances in Staphylococci of Different Origins and Species With Activity Against Relevant Pathogens. Front. Microbiol..

[16] Parthasarathy S., Shah S., Sager A.R., Rangan A., Durugu S. (2020). *Staphylococcus lugdunensis*: Review of Epidemiology, Complications, and Treatment. Cureus.

[17] Ho P.-L., Leung S.M.-H., Chow K.-H., Tse C.W.-S., Cheng V.C.-C., Tse H., Mak S.-K., Lo W.-K. (2015). Carriage niches and molecular epidemiology of *Staphylococcus lugdunensis* and methicillin-resistant *S. lugdunensis* among patients undergoing long-term renal replacement therapy. Diagn. Microbiol. Infect. Dis..

[18] van der Mee-Marquet N., Achard A., Mereghetti L., Danton A., Minier M., Quentin R. (2003). *Staphylococcus lugdunensis* Infections: High Frequency of Inguinal Area Carriage. J. Clin. Microbiol..

[19] Becker K., Heilmann C., Peters G. (2014). Coagulase-Negative Staphylococci. Clin. Microbiol. Rev..

[20] Sato M., Kubota N., Horiuchi A., Kasai M., Minami K., Matsui H. (2016). Frequency, clinical manifestations, and outcomes of *Staphylococcus lugdunensis* Bacteremia in children. J. Infect. Chemother..

[21] McHardy I.H., Veltman J., Hindler J., Bruxvoort K., Carvalho M.M., Humphries R.M. (2017). Clinical and Microbiological Aspects of β-Lactam Resistance in *Staphylococcus lugdunensis*. J. Clin. Microbiol..

[22] Hellbacher C., Törnqvist E., Söderquist B. (2006). *Staphylococcus lugdunensis*: Clinical spectrum, antibiotic susceptibility, and phenotypic and genotypic patterns of 39 isolates. Clin. Microbiol. Infect..

[23] Böcher S., Tønning B., Skov R.L., Prag J. (2009). *Staphylococcus lugdunensis*, a Common Cause of Skin and Soft Tissue Infections in the Community. J. Clin. Microbiol..

[24] Yen T.Y., Sung Y.J., Lin H.C., Peng C.T., Tien N., Hwang K.-P., Lu J.J. (2016). Emergence of oxacillin-resistant *Staphylococcus lugdunensis* carrying staphylococcal cassette chromosome *mec* type V in central Taiwan. J. Microbiol. Immunol. Infect..

[25] Kleiner E., Monk A.B., Archer G.L., Forbes B.A. (2010). Clinical Significance of *Staphylococcus lugdunensis* Isolated from Routine Cultures. Clin. Infect. Dis..

[26] Teh J.S.K., Pantelis I., Chen X., Sadlon T., Papanaoum K., Gordon D.L. (2022). Antimicrobial Susceptibility Testing for *Staphylococcus lugdunensis*. J. Clin. Microbiol..

[27] Liu M.C., Cao H., Lau A., Chow K.H., Lai E.L., Tse C.W., Wu A.K., Ho P.L. (2019). Structures of SCCmec elements in methicillin-resistant *Staphylococcus lugdunensis* are closely related to those harboured by community-associated methicillin-resistant Staphylococcus aureus. J. Med. Microbiol..

[28] Argemi X., Hansmann Y., Riegel P., Prévost G. (2017). Is *Staphylococcus lugdunensis* Significant in Clinical Samples?. J. Clin. Microbiol..

[29] Costello E.K., Lauber C.L., Hamady M., Fierer N., Gordon J.I., Knight R. (2009). Bacterial Community Variation in Human Body Habitats Across Space and Time. Science.

[30] Oh J., Byrd A.L., Kong H.H., Segre J.A., NISC Comparative Sequencing Program (2016). Temporal Stability of the Human Skin Microbiome. Cell.

