# Supplementary material for: Antimicrobial Resistance and Antimicrobial Activity of Staphylococcus lugdunensis Obtained from Two Spanish Hospitals
Source: Microorganisms. 2022 Jul 22;10(8):1480. doi: 10.3390/microorganisms10081480 (PMC9332302; doi:10.3390/microorganisms10081480)
Supplement: Supplementary file 1 [file microorganisms-10-01480-s001.zip › microorganisms-1806380-supplementary.pdf]

Supplementary material

Table S1. Clinical characteristics of the *S. lugdunensis* isolates included in the study.

Isolate	Origin[a]	Sex[b]	Age	Service	Hospital[c]
C10342	Blood	M	-	Infectious disease service	HSP
C10108	Blood	M	55	Cardiology	HRV
C10107	Blood	M	82	Internal Medicine/Traumatology	HRV
C10109	Blood	M	51	Emergency service	HRV
C9143	Blood	F	81	Emergency service	HSP
C9142	Blood	F	34	Emergency service	HSP
C9912	Blood	M	82	Emergency service	HSP
C9144	Blood	M	69	Emergency service	HSP
C9151	Catheter	M	57	Nephrology	HSP
C9148	Catheter	M	69	Nephrology	HSP
C9147	Catheter	M	53	Nephrology	HSP
C9161	Catheter	M	69	Nephrology	HSP
C9954	Catheter	M	57	Nephrology	HSP
C9982	Catheter	M	74	Nephrology	HSP
X700	Catheter	M	51	Nephrology	HSP
C9145	Catheter	F	73	Nephrology	HSP
X312	Catheter	M	57	Nephrology	HSP
X536	Catheter	F	75	Nephrology	HSP
C10341	Catheter	M	74	Nephrology	HSP
X313	Catheter	F	45	Home hospitalization	HSP
C9146	Catheter	M	54	Nephrology	HSP
C9925	Epidemiological	F	66	Internal Medicine/Traumatology	HRV
C10513	Genital Exudate	F	39	Primary care	HSP
C9149	Genital Exudate	F	41	Gynaecology	HSP
C9150	Genital Exudate	M	25	Primary care	HSP
X314	Genital Exudate	F	29	Gynaecology	HSP
C9343	SSTI	F	2	Emergency service	HSP
X463	SSTI	M	70	General Surgery	HRV
C10052	SSTI	F	93	Primary care	HSP
C10320	SSTI	F	58	Gynaecology	HSP
C9896	SSTI	M	76	Traumatology	HSP
C10511	SSTI	F	43	Gynaecology	HSP
C9894	SSTI	M	55	Traumatology	HSP
C10343	SSTI	M	50	Angiology and vascular surgery	HSP
C9342	SSTI	F	49	Primary care	HSP
C9895	SSTI	F	89	Internal Medicine	HSP
C10042	SSTI	F	38	Gynaecology	HSP
C10248	SSTI	F	46	Gynaecology	HSP
C10510	SSTI	M	40	Primary care	HSP
X462	SSTI	M	43	Emergency service	HRV
C9890	SSTI	M	67	Primary care	HSP
C10053	SSTI	F	24	Primary care	HSP

C9892	SSTI	F	51	Primary care	HSP
C9893	SSTI	M	72	Primary care	HSP
C9897	SSTI	M	72	Nephrology	HSP
C10112	SSTI	M	73	Primary care	HSP
C9891	SSTI	M	73	Emergency service	HSP
C9927	SSTI	F	54	Traumatology	HRV
C10113	SSTI	F	31	Emergency service	HSP
X508	Urine	M	89	Primary care	HSP
C10247	Urine	F	36	Primary care	HSP
C9980	Urine	F	81	Primary care	HSP
C9981	Urine	F	26	Primary care	HSP
C9889	Urine	M	76	Emergency service	HSP
C9159	Urine	M	89	Emergency service	HRV
C9911	Urine	F	25	General surgery	HSP

[a]Origin: SSTI: skin and soft tissue infection.
[b]Sex: M, male; F, female.
[c]Hospital: HSP, San Pedro Hospital; HRV, Royo Villanova Hospital.

Table S2. Characteristics of the 37 indicator bacteria used in this study for the screening of antimicrobial activity production in the collection of 56 *S. lugdunensis* isolates.

**Specie (no of strains)**	**Strain**	**Origin**	**Relevant Antimicrobial Resistance Phenotype/Genotype**
S. aureus **(4)**	C1570	Human	Methicillin/*mecA*[a]
	C7246	Human	Methicillin/*mec*C[a]
	C5313	Human	Methicillin/*mecA*
	ATCC29213 (C411)		
S. delphini **(1)**	C9459	Wild animal	
S. pseudintermedius **(12)**	C2381	Pet	Methicillin/*mecA*
	C3930	Pet	Methicillin/*mecA*
	C2382	Pet	Methicillin/*mecA*
	C8187	Human	MDR
	C2915	Human	
	C2912	Pet	
	C8189	Human	
	C3917	Human	
	C8188	Human	
	C8368	Human	
	C3007	Pet	
	C3468	Pet	
S. epidermidis **(1)**	C2663	Human	Methicillin/*mecA* and Linezolid
S. haemolyticus **(1)**	C2709	Human	Linezolid
S. lugdunensis **(6)**	C9927	Human	
	C9981	Human	
	C9159	Human	
	C10107	Human	
	C9954	Human	
	C9980	Human	
S. sciuri **(1)**	C9780	Wild animal	
Enterococcus casseliflavus **(1)**	C1232		
E. durans **(1)**	C1433		Vancomycin/*vanA*
E. faecalis **(1)**	C3735		Vancomycin/*vanB2*
E. faecalis**(1)**	ATCC29212 (C410)		
E. faecium **(1)**	C2321		Vancomycin/*vanA*
E. gallinarum **(1)**	C2310		
E. hirae **(1)**	C1436		Vancomycin/*vanA*
Listeria monocytogenes **(1)**	CECT4032 (C137)		
Micrococcus luteus	C157		
Escherichia coli **(1)**	ATCC25922 (C408)		
Pseudomonas aeruginosa **(1)**	C3282		

[a]The C1570 isolate belonged to ST398 and the C7246 isolates belonged to ST1945.

Table S3. Antimicrobial activity profile of the 23 *S. lugdunensis* isolates characterized as antimicrobial producers against the 37 indicator bacteria.

		Antimicrobial activity of the antimicrobial producer isolates[a]																						
					InterA-AP[b]											IntraA-AP[b]							RA-AP[b]	
**Specie of indicator bacteria[c]**	**UR[d]-reference**	**C9954**	**C9161**	**C9148**	**C10107**	**C9894**	**C9980**	**C9893**	**C9159**	**C9145**	**C9892**	**C10052**	**C9890**	**C9911**	**10343**	**C9142**	**C9146**	**C9147**	**C9151**	**10320**	**10341**	**10511**	**C9897**	**C9342**
**MR-SA**	**C1570**	2	0	0	1	0	0	0	1	0	1	1	2	0	0	2	1	1	0	0	0	0	0	0
	**C7246**	2	0	0	1	0	0	0	0	0	0	1	0	0	0	0	0	0	0	0	0	0	0	0
	**C5313**	3	0	0	2	0	2	0	2	1	0	0	0	0	0	0	0	0	0	0	0	0	0	0
**MS-SA**	**C411**	4	2	2	0	1	1	2	0	1	2	1	0	1	1	0	0	0	0	0	0	0	0	0
S. delfini	**C9459**	2	0	0	0	0	0	0	0	0	0	0	0	0	0	0	0	0	0	0	0	0	0	0
**MR-SP**	**C2381**	2	2	2	0	1	0	0	1	0	0	0	0	0	0	0	0	0	0	0	0	0	0	0
	**C3930**	2	2	1	0	0	0	0	0	0	0	0	0	0	0	0	0	0	0	0	0	0	0	0
	**C2382**	2	2	2	2	1	0	0	1	0	0	0	1	0	0	0	0	0	0	0	0	0	0	0
**MS-SP**	**C8187**	3	3	3	3	3	1	1	1	0	1	1	1	0	0	0	0	0	0	0	0	0	0	0
	**C2915**	3	3	3	3	3	3	1	1	1	1	1	3	0	0	0	0	0	0	0	0	0	0	0
	**C2912**	3	3	3	3	3	3	1	1	1	1	1	3	0	0	0	0	0	0	0	0	0	0	0
	**C8189**	3	3	3	3	3	3	1	1	1	1	1	3	0	0	0	0	0	0	0	0	0	0	0
	**C3917**	3	3	1	3	2	1	0	0	0	1	0	1	0	0	0	0	0	0	0	0	0	0	0
	**C8188**	3	3	3	3	3	3	1	1	0	1	1	2	0	0	0	0	0	0	0	0	0	0	0
	**C8368**	2	2	1	0	0	0	0	0	0	0	0	0	0	0	0	0	0	0	0	0	0	0	0
	**C3007**	3	3	3	1	2	1	1	1	0	1	1	1	0	0	0	0	0	0	0	0	0	0	0
	**C3468**	3	3	3	3	2	3	3	3	2	1	1	3	0	0	0	0	0	0	0	0	0	0	0
S. epidermidis	**C2663**	3	0	1	0	2	0	1	1	0	2	3	2	0	0	0	0	0	0	0	0	0	0	0
S. haemolyticus	**C2709**	1	0	1	0	0	0	0	0	0	0	0	0	0	1	0	0	0	2	1	1	1	0	0
S. lugdunensis	**C9927**	2	2	1	0	0	0	0	0	0	0	0	0	0	0	0	0	0	0	0	0	0	0	0
	**C9981**	0	0	0	0	0	0	0	0	0	0	0	0	0	0	0	0	0	0	0	0	0	0	0
	**C9159**	0	0	0	0	0	0	0	0	0	0	0	0	0	0	0	0	0	0	0	0	0	0	0

	**C10107**	0	0	0	0	0	0	0	0	0	0	0	0	0	0	0	0	0	0	0	0	0	0	0
	**C9954**	0	0	0	0	0	0	0	0	0	0	0	0	0	0	0	0	0	0	0	0	0	0	0
	**C9980**	2	2	0	0	0	0	0	0	0	0	0	0	0	0	0	0	0	0	0	0	0	0	0
S.sciuri	**C9780**	2	2	0	2	2	2	0	2	0	2	0	1	0	0	0	0	0	0	0	0	0	0	0
Enterococcus . casseliflavus	**C1232**	0	0	0	2	1	0	2	0	0	0	0	0	0	0	0	0	0	0	0	0	0	0	0
***E. durans van*A**	**C1433**	0	1	0	0	0	0	0	0	0	0	0	0	0	0	0	0	0	0	0	0	0	0	0
***E. faecalis van*B2**	**C3735**	1	0	0	0	0	0	1	0	0	0	0	0	0	0	0	0	0	0	0	0	0	0	0
E. faecalis	**C410**	2	1	0	1	0	0	0	1	0	0	0	0	0	0	0	0	0	0	0	0	0	1	0
***E.faecium van*A**	**C2321**	0	0	0	0	0	0	0	0	0	0	0	0	0	0	0	0	0	0	0	0	0	0	0
E. gallinarum	**C2310**	2	2	0	1	0	0	2	0	2	0	0	0	0	0	0	0	0	0	0	0	0	0	0
***E. hirae van*A**	**C1436**	2	0	0	0	0	0	0	0	0	0	0	0	0	0	0	0	0	0	0	0	0	0	0
Listeria monocytogenes	**C137**	1	0	0	0	0	0	0	0	0	0	0	0	0	0	0	0	0	0	0	0	0	0	2
Micrococcus luteus	**C157**	4	2	2	1	2	1	0	0	0	0	0	0	0	0	0	0	0	0	0	0	0	0	0
Escherichia coli	**C408**	0	0	0	0	0	0	0	0	0	0	0	0	0	0	0	0	0	0	0	0	0	0	0
Pseudomonas aeruginosa	**C3282**	0	0	0	0	0	0	0	0	0	0	0	0	0	0	0	0	0	0	0	0	0	0	0

[a]The numbers indicate the size of the inhibition halo against each indicator bacteria: 4, halo>10mm; 3, 9>halo>6 mm; 2, 5>halo>3, 1, halo<3; 0, no halo.

[b]Categories of antimicrobial activity: **InterA**, Interespecific Activity (antimicrobial activity against different groups of bacteria belonging to different genera, in addition to staphylococci); **IntraA,** Intraespecific Activity (antimicrobial activity against different species of staphylococci, but not against other genera); **RA**, Reduced Activity (antimicrobial activity against one bacterial group).

[c]Abbreviations: MR, methicillin resistant; MS, methicillin susceptible; CoPS, coagulase-positive *Staphylococcus*; CoNS, coagulase-negative *Staphylococcus*; SA: *S. aureus;* SP: *S. pseudintermedius.*

[d]UR: University of La Rioja.
